# Influence of animal pain and distress on judgments of animal research justifiability among university undergraduate students and faculty

**DOI:** 10.1371/journal.pone.0272306

**Published:** 2022-08-08

**Authors:** Eric P. Sandgren, Robert Streiffer, Jennifer Dykema, Nadia Assad, Jackson Moberg

**Affiliations:** 1 Pathobiololgical Sciences, School of Veterinary Medicine, University of Wisconsin-Madison, Madison, WI, United States of America; 2 Medical History and Bioethics, School of Medicine and Public Health, University of Wisconsin-Madison, Madison, WI, United States of America; 3 University of Wisconsin-Madison Survey Center, University of Wisconsin-Madison, Madison, WI, United States of America; University of Lincoln - Brayford Campus: University of Lincoln, UNITED KINGDOM

## Abstract

Acceptance of animal research by the public depends on several characteristics of the specific experimental study. In particular, acceptance decreases as potential animal pain or distress increases. Our objective in this study was to quantify the magnitude of pain/distress that university undergraduate students and faculty would find to be justifiable in animal research, and to see how that justifiability varied according to the purpose of the research, or the species to which the animal belonged. We also evaluate how demographic characteristics of respondents may be associated with their opinions about justifiability. To accomplish this goal, we developed and administered a survey to students and faculty at the University of Wisconsin-Madison. Our survey employed Likert-style questions that asked them to designate the level of animal pain or distress that they felt was justifiable for each of the following six purposes—animal disease, human disease, basic research, human medicine, chemical testing, or cosmetic testing. These questions were asked about five different species of animals including monkeys, dogs/cats, pig/sheep, rats/mice, or small fish. We used the data to establish a purpose-specific pain/distress scale, a species-specific pain/distress scale, and a composite pain/distress scale that, for each respondent, averaged the extent of justifiable pain/distress across all purposes and species. For purpose, students were more likely to choose higher levels of pain for animal disease research, followed by human disease, basic research, human medicine, chemical testing, and cosmetic testing. Faculty were more likely to choose the same level of pain for the first four purposes, followed by lower levels of pain for chemical and cosmetic testing. For species, students were more likely to choose higher levels of pain for small fish and rats/mice (tied), pigs/sheep and monkeys (tied), than for dogs/cats. For faculty, order from least to most justifiable pain/distress was small fish, rats/mice, pigs/sheep, then dogs/cats and monkeys (the latter two tied). Interestingly, exploratory factor analysis of the pain/distress scales indicated that when it comes to justifying higher levels of pain and distress, respondents identified two distinct categories of purposes, chemical and cosmetic testing, for which respondents were less likely to justify higher levels of pain or distress as compared to other purposes; and two distinct categories of species, small fish and rats/mice, for which respondents were more likely to justify higher levels of pain/distress than other species. We found that the spread of acceptance of animal research was much smaller when survey questions included pain/distress compared to when only purpose or species were part of the question. Demographically, women, vegetarians/vegans, and respondents with no experience in animal research justified less animal pain/distress than their counterparts. Not surprisingly, a lower level of support for animal research in general was correlated with lower justifiability of pain/distress. Based on these findings, we discuss the role of animal pain/distress in regulatory considerations underlying decisions about whether to approve specific animal uses, and suggest ways to strengthen the ethical review and public acceptance of animal research.

## Introduction

Public support for animal research has been reported to vary according to three principle elements: the purpose of the research [1–7], the kind of animal employed [1, 2, 4–10], and the extent of potential pain and/or distress experienced by the animals [1, 6, 8, 11–13]. Identifying how these elements of a specific study influence people’s views can guide efforts to incorporate the public’s moral values into the criteria used in the research review process. The animal research community has a strategic interest in such efforts because animal research that strays too far from what the public finds ethically acceptable provides attractive targets for animal activist campaigns [14, 15]. Thus, incorporating the public’s moral values can help insulate the research community from criticism and maintain public support [16]. There also are direct ethical reasons for such efforts when public funding is used to support animal research [17] and when the justification for the animal research appeals to benefits to the public [18]. Identifying public views toward animal research are among the key research questions proposed in “Developing a Collaborative Agenda for Humanities and Social Scientific Research on Laboratory Animal Science and Welfare,” published in 2016 [19].

Surveys are used commonly to explore ethical views about animal research. We have taken this approach to identify university undergraduate and faculty views on animal research [20, 21]. In the current report, we examine the role of animal pain or distress on views about animal research. As noted above, the extent of animal pain or distress is an important determinant of research approval. For example, Plous [1] and Henry and Pulcino [8] reported that psychology student support for animal research using several species was far less if the research procedures caused pain, injury, or death. Ormandy and colleagues reported decreased animal research support when invasive procedures were involved [13]. A review by Hagelin and colleagues [12] reported that, in general, survey questions containing the words death, pain, or suffering elicited less respondent support for animal research. Richmond [22] found that, among the 44.9% of their respondents who reported being totally against or having serious reservations about animal research, the leading objection to animal research was that it “causes pain and suffering and/or is cruel.” Ipsos MORI has been carrying out research on public attitudes towards animal research since 1999. They found lower approval ratings for painful research [23]. Also in 1999, their focus group work for the Medical Research Council concluded that “the groups were extremely concerned about basic animal welfare issues, and about preventing pain from being experienced by animals” [24]. A consistent finding among their more recent research is that the avoidance of unnecessary suffering is an important proviso among supporters of animal research and that a significant minority say they cannot support animal research because of animal welfare concerns [25]. Most recently, they have found that “public regard for animal welfare appears to be increasing” [26]. These findings are not surprising, given the connections that people often develop with animals of various species, and people’s general aversion to causing animals pain or harm [27, 28].

In addition to the importance of understanding the pubilc’s views on animal research, specific institutions that engage in animal research should have an understanding of the views of their local community. Students and faculty have an especially strong interest in the kinds of animal research that takes place at their own academic institution. We administered our survey to undergraduate students and faculty at the University of Wisconsin-Madison (UW-Madison). Few surveys have included both faculty and students, and most surveys of undergraduate students included only a selected subset of respondents (for example, undergraduate psychology majors). UW-Madison is a large public research university that supports an expansive animal research program. It also has been the target of protests by animal activists. Our overarching objectives in this study were to determine the importance of the issue of animal research to our academic community and to evaluate their knowledge about and trust in specific sources of information on this subject [20], to understand the dependence of judgements about research justifiability on experimental purpose and species [21], and to identify whether and how animal pain or distress influenced views of research justifiability. Below, we present findings and conclusions related to this latter objective by addressing the following specific questions: (i) how does experimental pain or distress influence respondents’ judgements about the justifiability of different research purposes; (ii) how does it influence perceived justifiability of use of different research species; and (iii) how does support of research involving animal pain or distress depend on respondent gender, academic discipline, student year or faculty rank, dietary preferences, and experience with animal research? The answers to these questions can help us formulate policy and direct university decisions about animal research.

## Materials and methods

We have described our survey, survey methodology, and respondent populations previously [20, 21]. Briefly, in fall of 2016, we administered the survey to 8,000 randomly selected undergraduate students (out of a total enrolled population of 29,536), and in spring of 2017 to all 2,153 faculty. Students were contacted by email and received up to three email reminders. Faculty were contacted by letter containing a $2-bill cash incentive and received up to three email and one written reminders. Responses were received from 782 undergraduate students, for a response rate of 9.8%, and from 942 members of the faculty, for a response rate of 44%. Although the student response rate is low, Fosnacht and colleagues [29] reported good data reliability in student surveys with 5–10% response rate as long as the sample size reached 500 respondents. The UW-Madison Educational and Social/Behavioral Sciences Institutional Review Board reviewed and approved this study.

Survey questions asked respondents for self-reports about the importance of animal research, how much they know about animal research and its regulation, what sources they trust to provide unbiased information on the subject, and about the justifiability of animal research for certain research purposes, for different kinds of animals, and with varying amounts of animal pain or distress. Finally, we asked questions to identify respondent’s general attitudes toward animal use by humans, whether they had been vegetarian or vegan, whether they had participated in animal research, and demographic characteristics including gender, academic discipline, year in school for students, and rank for faculty.

Statistical analyses employed STATA (Stata Corporation) using non-parametric tests, as the latter do not assume that dependent variables are normally distributed. These included Wilcoxon/Mann-Whitney and Kruskal-Wallis tests. We used an ordinal logistic regression model or logistical regression for dichotomous dependent variables. Significance is expressed as: *p<0.05, **p<0.01, ***p<0.001.

For questions that asked about animal pain or distress, we included an experiment to identify the effect of question wording on responses. Approximately equal numbers of respondents received questions containing the phrase “pain or distress” versus “physical or emotional pain or suffering”. Our hypothesis was that “pain or distress” would be associated with less respondent concern.

Limitations of the study include sample population limited to UW-Madison and no data collected on actual UW-Madison IACUC behavior to explore its conformity to the respondents’ views.

## Results

Respondent characteristics are described in Table 1.

**Table 1:** Categories and numbers of survey participants^1^.

Participant category	Student, n	Faculty, n
	%	%
**All, n**	782	942
**Demographics %**		
Gender		
Male	36.7	65.8
Female	61.6	31.1
Discipline		
Biological Science	44.5	38.4
Physical Science	20.2	19.6
Social Science	22.5	24.2
Arts and Humanities	7.2	17.8
Year in school		
Freshman	29.7	n/a
Sophomore	22.0	n/a
Junior	24.9	n/a
Senior	23.4	n/a
Faculty rank		
Assistant Professor	n/a	20.4
Associate Professor	n/a	19.4
Full Professor	n/a	60.2
**Experiences with Animals %**		
Dietary preferences, last 5 years		
Vegetarian/vegan	19.6	16.6
Not vegetarian/vegan	80.4	83.4
Experience animal research		
Done animal res. project	14.0	29.8
Not done an. res. project	86.0	70.2
**Support for animal research %**: “I do not think that there is anything wrong with using animals in medical research.”		
Agree	43.4	60.6
Neither agree nor disagree	21.7	18.6
Disagree	35.0	20.9

^1^From ref. [20]. Some respondents did not answer every survey question, so subcategories do not always add up to their population total.

### Pain/Distress (PD) scale

Specific survey questions are presented in Table 2.

**Table 2:** Survey questions.

Question	Question wording	Responses
AAS-6	To what extent do you agree or disagree with each of the following statements about the use of animals?	1 = Strongly agree; 2 = Agree; 3 = Neither agree nor disagree; 4 = Disagree; 5 = Strongly disagree
	[a] It is morally wrong to hunt wild animals for sport‡	
	[b] I do not think that there is anything wrong with using animals in medical research	
	[c] I think it is perfectly acceptable for cattle and hogs to be raised for human consumption	
	[d] It is unethical to breed purebred dogs when millions of dogs are killed in animal shelters each year‡	
	[e] I sometimes get upset when I see wild animals in cages at zoos‡	
	[f] Basically, humans have the right to use animals as we see fit	
ARAS-P	How often do you feel it is justifiable to use animals in research studies for each of the following purposes?	1 = Never; 2 = Rarely; 3 = Sometimes; 4 = Usually; 5 = Always
	[a] To look for ways to prevent or treat animal diseases	
	[b] To improve the production of livestock to lower the cost or raise the quality of agricultural products such as meat, milk, and eggs	
	[c] To conduct basic research to learn more about how organs, tissues, and cells function	
	[d] To look for ways to prevent or treat human diseases	
	[e] To test new medications for humans	
	[f] To test the safety of workplace or household chemicals	
	[g] To test the safety of cosmetics	
ARAS-S	How often do you feel it is justifiable to use each of the following types of animals in research studies?	1 = Never; 2 = Rarely; 3 = Sometimes; 4 = Usually; 5 = Always
	[a] Monkeys	
	[b] Dogs and cats	
	[c] Pigs and sheep	
	[d] Rats and mice	
	[e] Small fish such as minnows or zebrafish	
ARAS-PD^1^	If monkeys were used in research for each of the following purposes, what is the highest level of [physical or emotional pain or suffering/pain or distress] that would be justifiable to you?	1 = None; 2 = A little; 3 = Some; 4 = Quite a bit; 5 = A great deal
	[a] To look for ways to prevent or treat animal diseases	
	[b] To conduct basic research to learn more about how organs, tissues, and cells function	
	[c] To look for ways to prevent or treat human diseases	
	[d] To test new medications for humans	
	[e] To test the safety of workplace or household chemicals	
	[f] To test the safety of cosmetics	
	This question then was repeated for each of the following animals: dogs or cats, pigs or sheep, mice or rats, and small fish such as minnows or zebrafish.	
IVB	In the past 5 years, have you ever…	1 = Yes; 2 = No
	[a] been a vegetarian or vegan?	
	[b] worked on a research project that used animals?	

‡Reverse scored in AAS-6. Our 1–5 scale in the survey questions also reversed the order of agreement relative to Herzog and colleagues; see [21].

^1^ For species other than monkeys and dogs or cats, we also asked for the highest level of pain and/or distress respondents would justify for the purpose “To improve the production of livestock to lower the cost or raise the quality of agricultural products such as meat, milk, and eggs”, or, for fish, “To improve the production of fish for higher quality food products”. Because this purpose was not addressed in the pain or distress scale for all species, we excluded it from our ARAS-PD analyses.

As described in earlier reports [20, 21], we asked six questions to identify respondents’ general views on the use of animals by humans using a survey based on Herzog and colleagues’ Animal Attitudes Scale (AAS-6) [30, 31]. We also asked for respondents’ judgments of animal research justifiability based on scientific purpose (Animal Research Attitude Scale-Purpose, ARAS-P) and on species (Animal Research Attitude Scale-Species, ARAS-S) [21].

For each purpose and species, we also asked respondents to identify the highest level of pain and/or distress they would be willing to justify in a research experiment. This information formed the basis of our Animal Research Attitude Scale-Pain/Distress (ARAS-PD) (Table 2).

For each ARAS-PD question, approximately one-half of the respondents received text reading “physical or emotional pain or suffering” and the rest received text reading “pain or distress”. Differences in response will be discussed below, but, in general, responses with either wording were not significantly different, so data are pooled for most of the following results and discussion. We also asked about respondents’ dietary preferences and experience with animal research (Table 2, question IVB). To identify respondents’ general support for animal research, we separately pooled responses to the AAS-6 question [b] as “agree” (strongly agree and agree), “neither agree nor disagree”, or “disagree” (strongly disagree and disagree).

### Characteristics and validation of ARAS-PD

Data used to generate ARAS-PD scores are shown for students in Table 3 and for faculty in Table 4. Each entry represents the mean and standard deviation of responses to the questions’ 1–5 scales. Scores are proportional to the extent of justifiable animal research pain and/or distress reported by respondents for that purpose and species (higher numbers indicate more pain is justified).

**Table 3:** Student ARAS-PD scores as a function of research purpose and species (1–5 scale)^1^.

Purpose	Species				
	Small fish	Rat, mouse	Pig, sheep	Monkeys	Dog, cat
Animal disease	3.2 (1.3)	3.2 (1.3)	2.9 (1.2)	2.9 (1.1)	2.7 (1.2)
Human disease	3.1 (1.4)	3.2 (1.3)	2.8 (1.2)	2.9 (1.2)	2.6 (1.3)
Basic research	3.0 (1.3)	3.0 (1.3)	2.7 (1.2)	2.6 (1.2)	2.5 (1.2)
Human medicine	2.9 (1.4)	3.0 (1.4)	2.6 (1.2)	2.7 (1.2)	2.4 (1.2)
Chemicals	2.6 (1.4)	2.5 (1.4)	2.1 (1.2)	2.1 (1.1)	2.0 (1.2)
Cosmetics	2.2 (1.4)	2.1 (1.3)	1.8 (1.1)	1.7 (1.0)	1.6 (1.0)

^1^Data presented as mean (standard deviation).

**Table 4:** Faculty ARAS-PD scores as a function of research purpose and species (1–5 scale)^1^.

Purpose	Species					
	Small fish	Rat, mouse	Pig, sheep	Monkeys		Dog, cat
Animal disease	3.2 (1.1)	3.2 (1.1)	2.9 (1.1)		2.8 (1.0)	2.8 (1.1)
Human disease	3.3 (1.2)	3.2 (1.2)	3.0 (1.1)		2.9 (1.1)	2.8 (1.1)
Basic research	3.2 (1.2)	3.1 (1.1)	2.8 (1.0)		2.6 (1.0)	2.7 (1.1)
Human medicine	3.2 (1.2)	3.1 (1.2)	2.9 (1.1)		2.8 (1.1)	2.7 (1.1)
Chemicals	2.8 (1.3)	2.6 (1.2)	2.2 (1.0)		2.1 (1.0)	2.2 (1.1)
Cosmetics	2.1 (1.3)	2.0 (1.1)	1.7 (0.9)		1.6 (0.8)	1.6 (0.9)

^1^Data presented as mean (standard deviation).

We used this data to generate three pain/distress (PD) scales: Purpose PD scale, Species PD scale, and composite PD scale (Table 5). To calculate the ARAS-Purpose PD scores, we added the values in a row of Tables 2 or 3, then divided by the number of columns (i.e., 5) to normalize to a 1–5 scale. To calculate the ARAS-Species PD scores, we added the values in a column of Tables 2 or 3, then divided by the number of rows (i.e., 6). The ARAS-Composite PD scores represent the sum of all cells in a table divided by 30, again to normalize to a 1–5 scale. The composite score indicates for each respondent the overall extent of their acceptance of research pain/distress justifiability across all purposes and species. Cronbach’s Alpha for the composite PD scale approached 1, indicating good reliability. Exploratory factor analysis suggested 3-factor solutions (Table 6; S1 and S2 Tables). Based on orthogonal varimax rotation, the three factors together accounted for about 90% of the variance. For students, factor 1 was related to the species categories pigs and sheep, monkeys, and dogs and cats for all purposes. Factor 2 was related to small fish, rats and mice, and pigs and sheep for all purposes except chemicals and cosmetics. Factor 3 was related to chemicals and cosmetics for all species. Faculty showed a similar pattern, except factors 1 and 2 were reversed in order, which means faculty displayed slightly more variation compared to students in responses to purposes other than chemicals and cosmetics than they did to pigs and sheep, monkeys, and dogs and cats. These findings suggest that small fish and mice and rats are in a unique species-grouping of less concern to respondents when research involves pain, and that chemicals and cosmetics are in a unique purpose-grouping for which respondents justify much less pain than for other purposes.

**Table 5:** ARAS-PD purpose scale, species scale, and composite scale scores (1–5 scale).

Scale	Student	Faculty	Student vs.
	X±SD	X±SD	Faculty, p^1^
Purpose PD scale			
Animal disease	3.0 (1.1)	3.0 (1.0)	0.83 ns
Human disease	2.9 (1.2)	3.0 (1.0)	0.003**
Basic research	2.8 (1.1)	2.9 (1.0)	0.011*
Human medicine	2.7 (1.1)	3.0 (1.0)	<0.000***
Chemicals	2.2 (1.1)	2.4 (1.0)	<0.001***
Cosmetics	1.9 (1.0)	1.8 (0.9)	0.34 ns
Species PD scale			
Small fish	2.8 (1.2)	3.0 (1.1)	0.003**
Rat, mouse	2.8 (1.2)	2.8 (1.0)	0.51 ns
Pig, sheep	2.5 (1.0)	2.6 (0.9)	0.011*
Monkeys	2.5 (1.0)	2.5 (0.9)	0.68 ns
Dog, cat	2.3 (1.0)	2.5 (0.9)	<0.000***
Composite PD scale	2.6 (1.0)	2.7 (0.9)	0.053 ns
Cronbach’s Alpha	0.98	0.98	
EFA^2^ Eigenvalues			
Factor 1	18.8	20.3	
Factor 2	3.5	3.2	
Factor 3	1.9	1.8	

^1^All analyses are Wilcoxon/Mann-Whitney tests.

*p<0.05

**p<0.01

***p<0.001.

^2^EFA, exploratory factor analysis.

**Table 6:** Principal factor analysis and variance, orthogonal varimax rotation.

Respondents	Factor	Variance	Difference	Proportion	Cumulative
Students	1	10.6	3.4	0.40	0.40
	2	7.2	1.0	0.27	0.67
	3	6.3	-	0.23	0.89
Faculty	1	9.7	0.5	0.36	0.36
	2	9.2	3.2	0.34	0.70
	3	6.0	-	0.22	0.92

For students, the order of pain/distress justifiability for purpose, from high to low, was animal disease, human disease, basic research, human medicine, chemicals, and cosmetics; for species, the order was small fish and rat/mouse (tied), then pig/sheep and monkeys (tied), and finally dog/cat. Order for both purpose and species matched student order in the ARAS-P and ARAS-S scales, respectively [21]. For faculty, animal disease, human disease, human medicine, and basic research justified nearly equivalent pain/distress. Faculty scores for monkeys and dogs/cats were identical. For 4 of 6 purposes and 3 of 5 species, faculty scores were significantly higher than student scores, although the difference of composite scores was only near-significant (p = 0.053). Note that for both students and faculty, mean highest justifiable pain/distress fell between “a little” (score of 2) and “some” (score of 3), except for “cosmetics”.

To determine how the ARAS-Composite PD score was influenced by gender, academic discipline, student year in school, faculty rank, experience with animal research, and dietary choices, we performed multivariate analyses of our data (Table 7).

**Table 7:** ARAS-composite PD scale demographics.

	Students					Faculty					Students vs. Faculty		
Respondent characteristics	Mean	SD	Odds Ratio P		[95% CI]	Mean	SD	Odds Ratio P		[95% CI]	Odds Ratio P		[95% CI]
All	2.6	.98				2.7	.89						
Students vs (Faculty)											1.9	.049	[1.0, 3.5]
Gender													
(Male)	3.0	.94				2.8	.90						
Female	2.3	.89	.38	.000	[.25, .59]	2.4	.80	.52	.002	[.34, .78]	.58	.002	[.41, .82]
Division													
(Biological Sciences)	2.5	.87				2.8	.90						
Physical Sciences	2.8	1.0	1.1	.760	[.66, 1.8]	2.8	.82	.92	.674	[.63, 1.3]	.97	.887	[.68, 1.4]
Social Sciences	2.5	1.0	1.3	.390	[.72, 2.3]	2.6	.88	.90	.623	[.58, 1.4]	.92	.684	[.62, 1.4]
Humanities	2.4	1.1	1.2	.736	[.48, 2.9]	2.4	.89	.47	.002	[.34, .78]	.54	.007	[.34, .84]
Year in School													
(Freshman)	2.6	.97											
Sophomore	2.6	.98	1.1	.676	[.75, 1.6]								
Junior	2.5	.97	1.1	.729	[.75, 1.5]								
Senior	2.7	.99	1.4	.069	[.97, 2.0]								
Academic Rank													
(Assistant Professor)						2.6	.89						
Associate Professor						2.6	.90	.97	.888	[.68, 1.4]			
Full Professor						2.7	.89	1.0	.769	[.78, 1.4]			
Worked on Animal Research													
(Yes)	2.8	.97				2.8	.93						
No	2.5	.97	.65	.020	[.45, .93]	2.6	.88	1.1	.409	[.83, 1.6]	1.1	.474	[.82, 1.5]
Vegetarian or vegan													
(Yes)	2.1	1.0				2.2	.88						
No	2.7	.93	3.0	.000	[2.1, 4.2]	2.8	.87	2.9	.000	[2.1, 4.0]	2.7	.000	[2.0, 3.7]
Student vs Faculty Interactions													
Student X Female											.54	.002	[.36, .79]
Student X No An. Res.											.54	.013	[.33, .88]
Student X Not Veg./Vegan											1.1	.616	[.71, 1.8]
Model fit statistics													
N			737					925			1662		
Pseudo R2			.0227					.0155			.0186		
Log likelihood			-3181					-4013			-7276		

Gender had a strong effect on PD justifiability, and, among both students and faculty, women justified less PD than men, although the magnitude of the gender difference was significantly smaller for faculty. Responses based on student year in school or faculty rank did not differ significantly. Having participated in animal research significantly increased the reported extent of highest justifiable PD for students but not faculty. Dietary choices had a dramatic effect, and vegetarian/vegan students or faculty were 3.0 and 2.9 times less likely to justify PD than those who were not.

**Fig 1:**
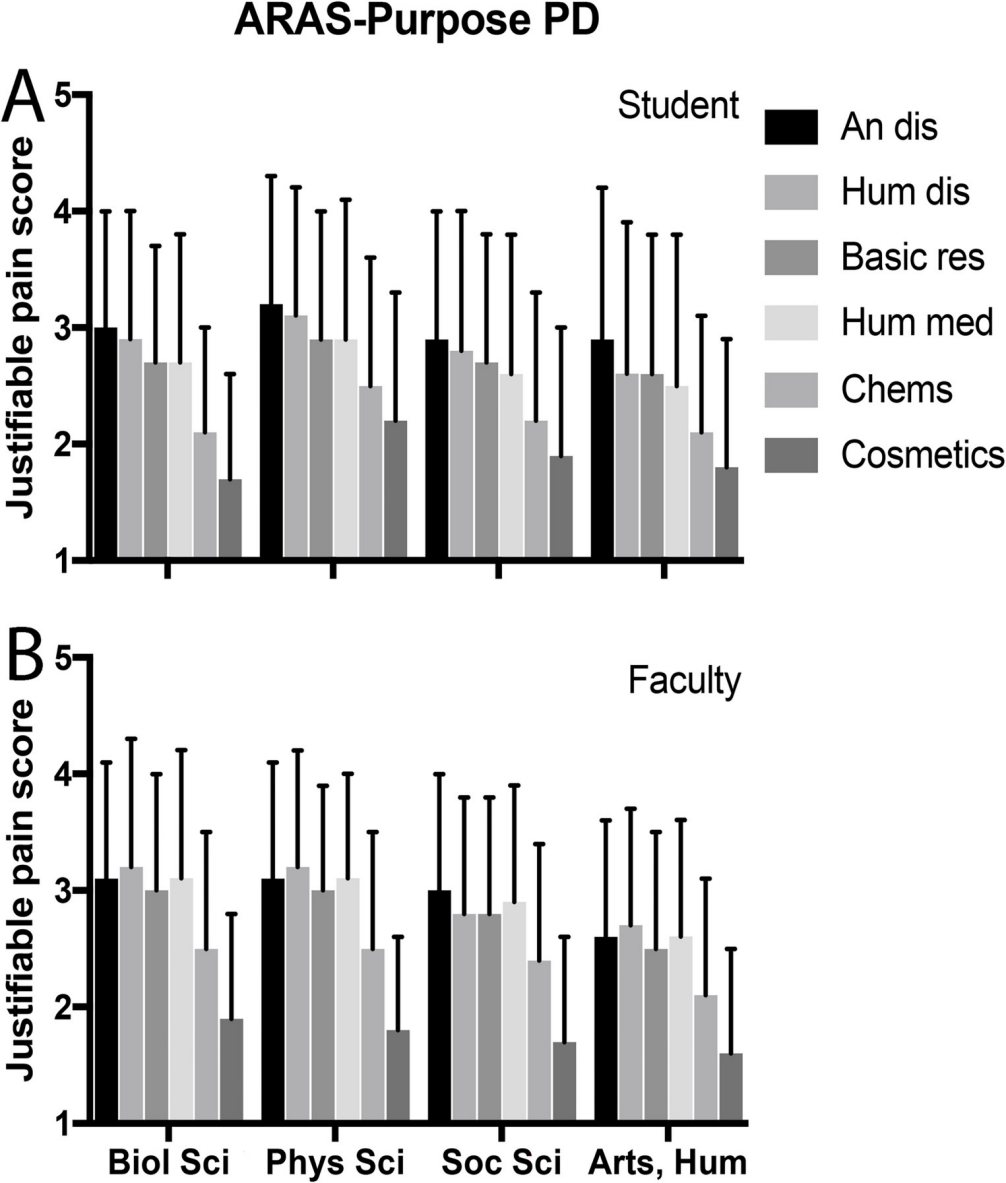
ARAS-purpose PD score versus discipline.

**Fig 2:**
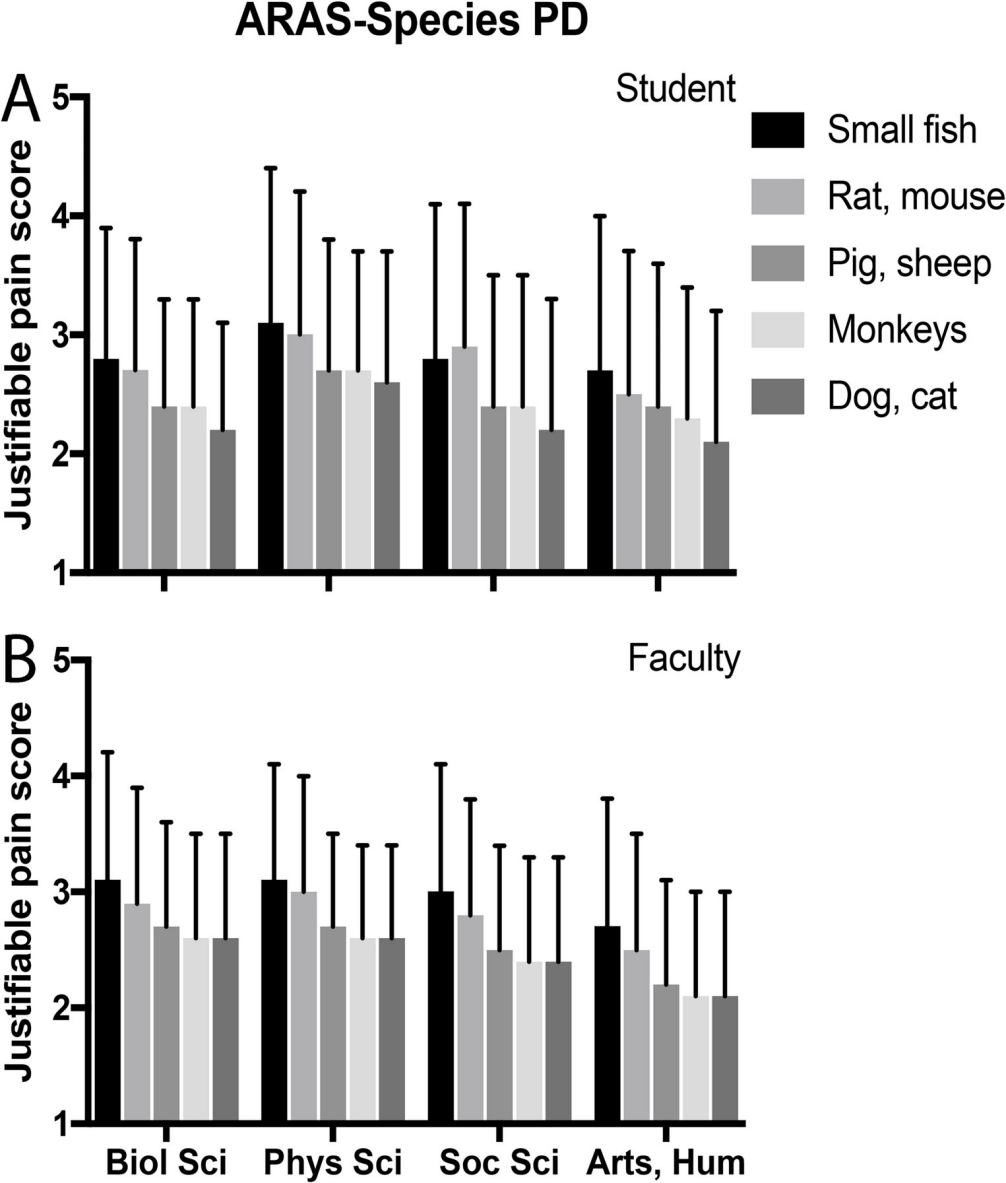
ARAS-species PD score versus discipline.

Discipline justifiability scores were highest for students in physical sciences compared to other disciplines, but this difference was not significant after multivariate analyses. Among faculty, only arts and humanities reported a significantly different (lower) justifiability than biological sciences. Figs 1 and 2 show variation by discipline of Purpose PD scores and Species PD scores for students and faculty, respectively. Orders of rankings varied slightly across disciplines.

Comparing students to faculty using multivariate analyses, gender and dietary experiences showed significantly different scores. First, students were slightly more likely (p = 0.049) to justify higher scores than faculty (the lower absolute mean score for students compared to faculty reflects the greater proportion of women among students). As noted, gender differences were smaller for faculty than for students, with males scoring higher for students compared to faculty, and females scoring lower for students compared to faculty. Absolute scores were the same between students and faculty in the physical sciences and in the arts and humanities, but higher for faculty than students in biological and social sciences. Significant interactions were identified between student and female, indicating that female students were 0.54 times as likely to justify higher pain or distress than female faculty; and between student and no animal research, indicating that, among those who had not participated in animal research, students were also 0.54 times as likely to justify higher pain or distress than faculty.

Finally, we evaluated the correlation between preexisting support of animal research and highest justifiable pain/distress scores (Table 8). Justifiability varied dramatically depending on respondents’ support for animal research, with student and faculty supporters responding on average at or above “some” (score of 3), while non-supporters on average would justify only “a little” (score of 2).

**Table 8:** ARAS-composite PD score versus responses to survey question AAS-6b, “I do not think that there is anything wrong with using animals in medical research”.

Response	Student Scores	Faculty Scores
	X (SD)	X (SD)
Agree or strongly agree	3.2 (0.9)	3.0 (0.8)
Neither agree nor disagree	2.6 (0.7)	2.5 (0.8)
Disagree or strongly disagree	1.9 (0.8)	2.0 (0.8)
p^1^	244***	159***

^1^Wilcoxon/Mann-Whitney test.

***p<0.001.

### Influence of question word choice on ARAS-PD

We tested the hypothesis that questions asking respondents to consider animal wellbeing would receive greater approval if they contained the words “pain or distress” versus “physical or emotional pain or suffering”. We believed that the greater detail and the mention of suffering in the latter would evoke more concern. Approximately half of all respondents received questions with one phrase, and the rest received the other. Out of 33 questions linking purpose, species, and pain, students’ scores trended higher for “pain/distress” (more pain justified) for 30. Unexpectedly, faculty scores trended lower for all 33. However, no differences were significant for students by Chi square (p<0.05), and only three were significant for faculty. Neither student nor faculty differences in response to the two phrases were of high magnitude (student maximum 0.12 and faculty maximum 0.17 on the 1–5 scale). Based on the no-to-minimal response differences to question wording, respondents were pooled within student and within faculty populations for all other analyses.

## Discussion

Our data allow us to describe in more detail how the relationships between experimental purpose, animal species, and the extent of pain or distress influence judgments of animal research justifiability. We reported previously that, when pain or distress are not taken into account, respondents’ justifiability scores vary significantly according to research purpose and research species [21]. Interestingly, mean species justifiability is lower than mean purpose justifiability. Thus, respondents report less comfort with animal research justifiability when the question is asked in the context of species rather than purpose. This makes sense given that questions framed in terms of research purpose presumably make salient the most obvious reasons in favor of the research whereas questions framed in terms of species presumably make salient the individuals upon whom the burdens of research will be imposed. For both purpose and species scales, exploratory factor analysis (EFA) indicated one factor solutions [21], suggesting that a single factor accounted for most of the variance in response.

In the present report, we also evaluated how purpose and species influence the highest level of experimental pain or distress that respondents find justifiable. Several interactions became apparent. First, EFA indicated a three-factor solution when pain or distress were considered. This analysis identified purpose and species subcategories. Among purposes, chemical and cosmetic testing justified far less pain or distress than other purposes. Thus, with respect to pain or distress, we can classify the remaining purposes (animal disease, human disease, human medicine, basic research) as “legitimate purposes”. Pigs and sheep, dogs and cats, and monkeys represent a more “protected” group for which less pain was justifiable. A caveat is that when only legitimate purposes are taken into account, pigs and sheep join the group consisting of small fish and rats and mice as being of less concern to respondents.

Second, we observed that the spread of scores across both purpose and species was smaller when pain or distress was explicitly brought into consideration. For example, when students are asked how often animal research is justified for different kinds of research purposes, mean purpose justifiability falls between scores of 2.1 for cosmetics to 4.1 for animal disease, for a spread of 2.0 on the 1–5 scale (Table 9). For faculty, the spread is 1.8. However, when respondents are asked how much pain or distress is justified for different kinds of research purposes, the spreads of justifiable pain shrink to 1.1 and 1.2, for students and faculty respectively. For species, spreads of 1.2 for students and 1.8 for faculty shrink to 0.5 and 0.5 when pain or distress figure explicitly in the question. Thus, whereas scores have relatively broad spreads for animal research justifiability based only on purposes (ARAS-P) or species (ARAS-S), if pain or distress are explicitly mentioned, justifiability becomes much less variable across both purposes (ARAS-Purpose PD) and species (ARAS-Species PD).

**Table 9:** Justifiability score midpoints and ranges for ARAS-PD scales compared to ARAS-P and ARAS-S.

Scale		Range of scores	
		Students	Faculty
Purpose^1^		2.0	1.8
Purpose PD		1.1	1.2
Purpose, Legitimate		0.7	0.3
Purpose PD, Legitimate		0.3	0.1
Species		1.2	1.8
Species PD		0.5	0.5

^1^[21].

Differences among scale ranges are even more striking when we recalculate purpose scales to include only legitimate purposes (Table 9). The spread of purpose justifiability (ARAS-P, Legitimate) scores drops to 0.7 for students and 0.3 for faculty, but even further for pain/distress scales (ARAS-Purpose PD, Legitimate), to 0.3 and 0.1, respectively. Thus, for both students and faculty, the self-reported justifiability of animal research in response to questions that mention pain or distress is more restrictive compared to purpose or species justifications when pain or distress are not mentioned. After pain/distress, species is the next most important justification consideration, followed by purpose. Overall, our findings are consistent with other reports that perceived animal research justifiability decreases with increasing levels of animal pain or distress [1, 6, 8, 11–13]. Furthermore, our findings extend those reports to support the novel conclusion that, for many, considerations of pain and distress place more restrictive and less flexible constraints on justifiable animal research than other factors.

Our third specific research question asked about the relationship between pain or distress justifiability and respondent demographics. Our findings suggest the following. For students, gender has the strongest influence on ARAS-PD, with women displaying lower scores (less justifiability). For faculty, this gender difference remains but is smaller. Being vegetarian or vegan had the strongest effect (less justifiability) for faculty and was second strongest for students. For students only, experience with the practice of animal research increased pain/distress justifiability. Gender [1, 2, 6, 8, 12, 30, 32–38] and diet [13, 31, 39] similarly have been reported by others to influence animal research attitudes. Across disciplines, student scores were similar, but there was a difference among faculty: arts and humanities faculty scored significantly lower than biological sciences, while physical and social sciences were statistically not different than biological sciences. In an earlier publication [20], we reported that large majorities of all disciplines except biological sciences expressed little to no knowledge about animal research justifications or regulatory requirements, so the PD score similarities/differences do not merely reflect extent of self-reported knowledge.

Overall, student and faculty composite score means differed from one another, but at a high p value (p = 0.049), although, as noted in results, biological and social sciences faculty scores were higher than for the respective student disciplines. Previously, we reported that student-faculty differences in ARAS-P and ARAS-S also were largely the result of respondents in biological and social sciences [21]. Thus, these two disciplines seem to drive the differences between student and faculty animal research justifiability, while students and faculty in physical sciences and the arts and humanities do not differ from one another.

Previous reports indicate that question wording can affect responses to surveys about animal research justifiability [12]. We hypothesized that “pain or distress” would evoke less concern than “physical or emotional pain or suffering” and would elicit greater approval. Although the composite differences were not significant (p>0.05), both students and faculty displayed trends in the direction of their responses, with students trending towards expressing greater concern with the wording “physical or emotional pain or suffering”, and faculty trending in the opposite direction. We have no explanation for this difference.

### Consequences for review and approval of animal research

In the United States, the oversight of animal research is typically the responsibility of Institutional Animal Care and Use Committees (IACUCs), which are mandated by the Animal Welfare Act (AWA) (and its associated regulations) [40], and, for research funded by the Public Health Service (PHS), by the PHS Policy on Humane Care and Use of Laboratory Animals [41]. The PHS Policy covers all vertebrate species, while the AWA cover all warm-blooded animals, excluding farm animals used for agricultural research and mice, rats, and birds bred for use in research. In what ways do these laws, regulations, and polices (collectively: “public policy”) conform to or deviate from the views expressed by our respondents?

First, the AWA excludes entirely from its protections all cold-blooded animals as well as farm animals used for agricultural research, and mice, rats, and birds bred for research. Even though our respondents expressed *less* concern for research that causes pain in small fish, mice, and rats (we did not ask about birds or distinguish between mice and rats bred for research and wild mice and rats), the mean highest justifiable pain/distress for animals we did ask about still fell between “a little” and “some” (except for “cosmetics, which was slightly less than “a little”). The AWA thus fails to reflect our respondents’ concerns for these animals, a significant departure given that an estimated 90–95% of vertebrate animals used in research fall into these exempted categories. The PHS Policy, on the other hand, covers all vertebrates, and it should be noted that some institutions (such as the University of Wisconsin-Madison) voluntarily apply the PHS Policy to research not funded by the PHS.

For research involving covered animals, we outline below how public policy mandates that IACUC’s decisions be based primarily on (a) the application of the “3Rs”—to the extent consistent with sound scientific design that can answer the scientific question, *replace* animals with non-animal research modalities, *reduce* the number of animals used, and *refine* procedures so that they cause the least pain possible—and (b) a harm-benefit test, with approval requiring a positive balance of potential benefits to humans or animals versus harm to the animals used as subjects [41–44].

To receive PHS funding, institutions must follow the “US Government Principles for the Utilization and Care of Vertebrate Animals Used in Testing, Research, and Training” [45]. Principle II requires that procedures be undertaken with “due consideration of their relevance to human or animal health, the advancement of knowledge, or the good of society.” Although the wording does not explicitly state it, this requirement for “due consideration” appears to necessitate a harm-benefit test. Principle III mandates replacement and, when alternatives do not exist, reduction. Principles IV-VI mandate refinement through pain and distress minimization, use of sedation, analgesia, or anesthesia when appropriate, and painless euthanasia when there is unrelievable pain or distress. Principles VII and VIII mandate provision of appropriate living conditions and veterinary oversight, and appropriate personnel training, in part to minimize animal discomfort.

The AWA requires that IACUCs consider essentially these same issues, typically through a protocol form that provides information about the proposed research, including the following: the justification for using animals, for the particular species being proposed, and for the number of animals being requested; possible alternatives that could address the experimental question; whether the research is unnecessary duplicative; anesthesia, pain relief, and postsurgical care; humane endpoints and method of euthanasia; and living conditions [40, 46]. Although the AWA does not explicitly mandate a harm-benefit test, there would be little point in requiring some of the information it requires (especially the justification for the use of animals) if not for just such a purpose [47].

Finally, the *Guide for the Care and Use of Laboratory Animals*, which must be followed for PHS-funded animal research or for research taking place at AAALAC accredited facilities, states that for studies involving unrelieved pain or distress or other animal welfare concerns, “the IACUC is obliged to weigh the objectives of the study against potential animal welfare concerns” [41].

Thus, public policy encompasses the 3Rs together with a harm-benefit test. There is therefore a focus on avoiding unnecessary pain and distress to animals and ensuring that any remaining harm is justified by the potential benefits. This aspect of public policy, in broad terms, is consistent with the survey responses reported here.

However, even restricting our attention to research using covered species, two features of public policy give IACUCs broad discretion to interpret and apply public policy in ways that depart dramatically from the views of our respondents.

First, public policy authorizes IACUCs to make exceptions to almost any specific requirement. (The sole exception appears to be the AWA’s requirement to only use paralytics with anesthesia [40].) US Government Principle IX authorizes IACUCs to grant exceptions to any of the other principles, so long as the decision to do so is made with “due regard” to Principle II and is not granted solely for teaching or demonstration purposes. PHS Policy allows for deviations from the *Guide* whenever an “acceptable justification for a departure is presented” [41]. After discussing the wide range of issues that fall under an IACUC’s responsibility, the *Guide* acknowledges the possibility of exceptions to its standards on any of those issues, with the only caveat being that such exceptions “should be clearly defined and evaluated by the IACUC” [41]. And the AWA says “The IACUC shall determine that the proposed activities are in accordance with this subchapter *unless acceptable justification for a departure is presented in writing*…” [40] (emphasis added). The key phrases used in explaining the basis on which IACUCs are to exercise their exception-granting authority—“due regard,” “due consideration,” and “acceptable justification”—are largely undefined except that exceptions must be based on scientific considerations rather than considerations of cost-savings or teaching or demonstration needs [48].

Thus, almost all of the “requirements” need not be followed so long as the researcher has appropriate IACUC approval, and what counts as “appropriate” is left almost entirely unspecified. Any amount of pain, distress, or suffering, the use of cages and diets that are not species-appropriate, the withholding of postsurgical pain-relieving medications, and even the use of euthanasia methods that the American Veterinary Medical Association’s Panel on Euthanasia has found to be incompatible with a humane death, are all consistent with public policy so long as they are accompanied by a written scientific justification that has been approved by the relevant IACUC.

Second, IACUCs are granted decision-making discretion by virtue of the lack of any restrictions on the relative weights IACUCs can assign to different harms and benefits in its harm-benefit test. An IACUC could, consistently with public policy, decide that a remote chance of avoiding a minor inconvenience to a small number of humans outweighs causing extreme and long-lasting pain and suffering to a large number of animals. This means that IACUCs could use their discretion to approve research that would fall far outside the limits on justifiable pain and distress reported by our respondents. Conversely, this also means that IACUCs could, in theory, use their discretion to *prevent* research that falls within the limits of justifiable pain and distress reported by our respondents, or to provide *equal or greater* protections from pain and distress to, say, mice used in PHS-funded research as compared to nonhuman primates.

There appears to be little systematic research on how IACUCs actually exercise this discretion [49], and the existing research is conflicting. In 1989, Dresser found evidence that committees “had a strong commitment to reducing laboratory animal pain and distress” but also that “evaluating the justification for animal use remains problematic for committees” [48]. A follow-up review of protocol forms revealed that only 9 out of 24 asked researchers to provide a description of the study’s “potential value to society” [50]. In 1997, Borkowski and colleagues reported that 35% of the IACUC chairpersons who responded to their survey said that IACUCs should not assess scientific merit [51]. In 2001, Plous and Herzog [52] found low intercommittee agreement on whether a given protocol should be approved as written, contingently approved, deferred, or disapproved (kappa = -0.04, P = .32). This low level of agreement persisted even for protocols involving highly invasive and painful procedures. They also found low interrater agreement among IACUC members on the same committee (interclass correlation coefficient = 0.28 (P < .001)). In 2002, Graham [53] reported that 14% of the IACUC members they surveyed believed that IACUCs should not perform any scientific merit assessment. Graham also reported that roughly as many IACUC members in their survey agreed as disagreed that scientific merit and the search for alternatives to animal use should be more diligent for research involving nonhuman primates than for research involving rats or mice.

More recently, Silverman et al. conducted an analysis in 2015 of 87 transcribed protocol discussions at 10 IACUCs [54]. While pain and distress were the most often-mentioned topics, 33% of those mentions did not rise to the level of “a minimally effective discussion”. The issue of the social or scientific importance of the scientific question addressed by the research was only mentioned in 34.5% of the discussions and none of the 10 IACUCs conducted “any specific harm-benefit analysis”.

Two of the current report’s authors (EPS and RS) have been longstanding members and chairs of UW-Madison IACUCs, and recognize that the US Government Principles, PHS Policy, and the AWA do guide IACUC deliberations. Nevertheless, our experience also has shown us that the process of review of applications to use animals in research, teaching, or outreach would be improved to the extent that potential animal harm or distress can be quantified in each application. Brønstad and colleagues [42] and Keubler et al. [55] have discussed approaches to measuring research-associated animal harm. The authors point to a need to develop a better categorization of severity classifications and stronger evidence for measuring the extent of harm. (Unfortunately, a retrospective harm-benefit analysis indicates that even existing harm-benefit guidelines are not followed in some studies [44]). At the present time, however, recommendations to include even this level of quantification [43] have not been adopted in the United States and implementation in the European Union remains problematic [56]. As noted above, without such quantification, review remains qualitative and largely subjective between IACUCs [52], and even within a single IACUC, especially over time as membership changes. To provide consistency and to preserve valuable IACUC deliberation time, it would be prudent to develop and publish a system of harm severity categorization at the national and international levels. Guidelines then could be refined to fit specific research situations by institutional veterinary staff or others prior to IACUC review so that resulting IACUC decisions were more consonant with the views of the public.

Such systems would allow more systematic study of how IACUCs use the discretion noted earlier and, if necessary, could be used to enact restrictions at various levels that would help ensure IACUCs do not use their discretion in ways that departed dramatically from the expectations of the general public or more local stakeholders.

In summary, our survey results indicate that university undergraduate students and faculty set limits on the pain or distress justifiable in an animal research study. If used during the review of proposed animal research, national and international guidelines for measuring extent of pain and distress, coupled with consideration of animal species and research purpose, will strengthen the harm-benefit balancing test that should form the basis of current study approval decisions.

## Supporting information

S1 Table Student principal factor analysis, rotated factor loadings (0.5 or greater). (DOCX)

S2 Table Faculty principal factor analysis, rotated factor loadings (0.5 or greater. (DOCX)
