# Supplementary material for: Influence of animal pain and distress on judgments of animal research justifiability among university undergraduate students and faculty
Source: PLoS One. 2022 Aug 8;17(8):e0272306. doi: 10.1371/journal.pone.0272306 (PMC9359541; doi:10.1371/journal.pone.0272306)
Supplement: S1 Table — (DOCX) [file pone.0272306.s001.docx]

S1 Table. Student principal factor analysis, rotated factor loadings (0.5 or greater).

| Variable | | Factor 1 | Factor 2 | Factor 3 |
| --- | --- | --- | --- | --- |
| Species | Purpose |  |  |  |
| Small fish | Animal disease | 0.85 | - | - |
|  | Human disease | 0.87 | - | - |
|  | Basic research | 0.86 | - | - |
|  | Human medicine | 0.86 | - | - |
|  | Chemicals | 0.76 | - | - |
|  | Cosmetics | 0.64 | - | 0.61 |
| Rat, mouse | Animal disease | 0.81 | - | - |
|  | Human disease | 0.85 | - | - |
|  | Basic research | 0.82 | - | - |
|  | Human medicine | 0.84 | - | - |
|  | Chemicals | 0.70 | - | 0.52 |
|  | Cosmetics | 0.56 | - | 0.69 |
| Pig, sheep | Animal disease | 0.57 | 0.65 | - |
|  | Human disease | 0.62 | 0.64 | - |
|  | Basic research | 0.56 | 0.63 | - |
|  | Human medicine | 0.59 | 0.60 | - |
|  | Chemicals | - | - | 0.71 |
|  | Cosmetics | - | - | 0.87 |
| Monkeys | Animal disease | 0.52 | 0.64 | - |
|  | Human disease | 0.59 | 0.62 | - |
|  | Basic research | - | 0.60 | - |
|  | Human medicine | 0.50 | 0.63 | - |
|  | Chemicals | - | - | 0.72 |
|  | Cosmetics | - | - | 0.86 |
| Dog, cat | Animal disease | - | 0.77 | - |
|  | Human disease | - | 0.76 | - |
|  | Basic research | - | 0.76 | - |
|  | Human medicine | - | 0.72 | - |
|  | Chemicals | - | 0.53 | 0.72 |
|  | Cosmetics | - | - | 0.87 |
