# Supplementary material for: Influence of animal pain and distress on judgments of animal research justifiability among university undergraduate students and faculty
Source: PLoS One. 2022 Aug 8;17(8):e0272306. doi: 10.1371/journal.pone.0272306 (PMC9359541; doi:10.1371/journal.pone.0272306)
Supplement: S2 Table — (DOCX) [file pone.0272306.s002.docx]

S2 Table. Faculty principal factor analysis, rotated factor loadings (0.5 or greater).

| Variable | | Factor 1 | Factor 2 | Factor 3 |
| --- | --- | --- | --- | --- |
| Species | Purpose |  |  |  |
| Small fish | Animal disease | - | 0.84 | - |
|  | Human disease | - | 0.86 | - |
|  | Basic research | - | 0.86 | - |
|  | Human medicine | - | 0.85 | - |
|  | Chemicals | - | 0.76 | - |
|  | Cosmetics | - | 0.55 | 0.66 |
| Rat, mouse | Animal disease | 0.57 | 0.71 | - |
|  | Human disease | 0.56 | 0.76 | - |
|  | Basic research | 0.54 | 0.74 | - |
|  | Human medicine | 0.55 | 0.75 | - |
|  | Chemicals | - | 0.64 | 0.50 |
|  | Cosmetics | - | - | 0.72 |
| Pig, sheep | Animal disease | 0.71 | 0.50 | - |
|  | Human disease | 0.74 | 0.56 | - |
|  | Basic research | 0.70 | 0.51 | - |
|  | Human medicine | 0.72 | 0.54 | - |
|  | Chemicals | - | - | 0.64 |
|  | Cosmetics | - | - | 0.89 |
| Monkeys | Animal disease | 0.76 | - | - |
|  | Human disease | 0.80 | - | - |
|  | Basic research | 0.77 | - | - |
|  | Human medicine | 0.77 | - | - |
|  | Chemicals | 0.50 | - | 0.66 |
|  | Cosmetics | - | - | 0.84 |
| Dog, cat | Animal disease | 0.76 | - | - |
|  | Human disease | 0.79 | - | - |
|  | Basic research | 0.78 | - | - |
|  | Human medicine | 0.75 | - | - |
|  | Chemicals | 0.51 | - | 0.67 |
|  | Cosmetics | - | - | 0.88 |
